# Identification of Advantaged Genes for Low-Nitrogen-Tolerance-Related Traits in Rice Using a Genome-Wide Association Study

**DOI:** 10.3390/ijms26125749

**Published:** 2025-06-16

**Authors:** Zhiyuan Zhang, Laiyuan Zhai, Yuzhuo Liu, Lin Tian, Shuangbing Zhu, Congcong Shen, Juqing Jia, Kai Chen, Jianlong Xu

**Affiliations:** 1 College of Agriculture, Shanxi Agricultural University, Taigu 030801, China; kanshantea@163.com (Z.Z.); jiajuqing@sxau.edu.cn (J.J.); 2 Shenzhen Branch, Guangdong Laboratory for Lingnan Modern Agriculture, Agricultural Genomics Institute at Shenzhen, Chinese Academy of Agricultural Sciences, Shenzhen 518120, China; zhailaiyuan@caas.cn (L.Z.); zhushuangbing@caas.cn (S.Z.); shencongcong@caas.cn (C.S.); 3 State Key Laboratory of Crop Gene Resources and Breeding, Institute of Crop Sciences, Chinese Academy of Agricultural Sciences, Beijing 100081, China; 4 College of Agriculture, Shenyang Agricultural University, Shenyang 110866, China; 2023200066@stu.syau.edu.cn (Y.L.); tl369306@163.com (L.T.)

**Keywords:** rice, lower nitrogen, genome-wide association study (GWAS), haplotype analysis, candidate gene

## Abstract

Nitrogen is a crucial element that impacts rice yield and its constituent factors. The effects of reduced nitrogen levels on yield constitute is a complex quantitative trait that is controlled by multiple genes, and its genetic basis requires further exploration. In this study, 562 MAGIC line population and 284 germplasm varieties were used for genome-wide association analysis (GWAS) and haplotype analysis, aiming to detect quantitative trait loci (QTL) and candidate genes associated with tolerance to low nitrogen levels. The ratio of effective panicle number per plant (REPN), total number of grains per panicle (RTGN), seed setting rate (RSSR), thousand grain weight (RTGW), biomass (RBM), harvest index (RHI), and grain yield per plant (RGY) of low to normal nitrogen conditions were measured in this study. The RBM and RHI were directly closely related to RGY, while the RSSR indirectly and positively affected RGY through RHI, and the REPN and RTGN mainly indirectly and positively affected RGY through RBM. *LOC_Os06g06440* was the most likely gene affecting low-nitrogen-tolerance-related traits in rice within the region, ranging from 2.898 Mb to 3.046 Mb (148 kb) on chromosome 6, and the haplotype AA, with a significantly larger mean RGY of 0.95 and 1.53 in the MAGIC and germplasm varieties, respectively, was the advanced allele of *LOC_Os06g06440*. Nine *xian* (*indica*) varieties (IRIS_313-11624, IRIS_313-10932, CX382, B067, B249, IRIS_313-8215, IRIS_313-10544, B052, and B233) carrying the superior haplotype (AA) of *LOC_Os06g06440* and having a higher RGY were selected for the molecular marker-assisted selection of low nitrogen tolerance in rice. These results will enhance our knowledge of the genetic basis of tolerance to low levels of nitrogen and provide valuable information for improving tolerance to low levels of nitrogen in rice-breeding programs.

## 1. Introduction

Rice (*Oryza sativa* L.) is the main food crop for more than half of the world’s population, and its high and stable yield is essential to ensure food security. Nitrogen (N) is the core nutrient element affecting rice growth and development; it contributes to chlorophyll synthesis, energy metabolism, protein construction and other key physiological processes. Assuming that the world’s population will reach 10 billion by the year 2050, the demand for nitrogen in rice production will increase by 44%, and since crops use only 30–40% of applied nitrogen, the remaining 60–70% of unused nitrogen in agriculture is causing serious environmental and health problems [1]. The overapplication of nitrogen fertilizer is a common problem in rice production, which not only leads to low nitrogen utilization rates (usually less than 40%) but also causes environmental problems such as soil acidification, water eutrophication, and greenhouse gas emission. Therefore, in order to reduce resource waste, avoid pollution and achieve sustainable development, optimizing the use of nitrogen, tapping the genetic potential of rice varieties with low nitrogen tolerance and cultivating nitrogen-efficient varieties have become important research directions in order to achieve sustainable agricultural development.

The cloning of low-nitrogen-tolerant genes is the key to the breeding of low-nitrogen-tolerant varieties. With the achievements of the Rice Genome Project, many important genes and quantitative trait loci (QTL) have been discovered by genome-wide association studies (GWAS) [2], such as *OsGS1;1* [3], *OsTCP19* [4], *OsNPF6.1* [5], and *OsNLP4* [6], which provides great help for rice nitrogen utilization of the study on the properties and tolerance to low nitrogen. Among them, the *OsGS1;1* located on chromosome 2 encodes glutamine synthase, which is induced under low nitrogen conditions to affect grain formation by regulating glucose metabolism [3]. *OsTCP19* regulates the transcriptional response of nitrogen and targets *DWARF* and *LOW-TILLERING* (*DLT*) genes that promote tillering, affecting nitrogen utilization and tillering in rice [4]. The genome-wide association analysis of a diverse rice population revealed extreme nitrogen-related phenotypes over three successive years in the field, and researchers were able to identify an elite haplotype of nitrate transporter OsNPF6.1 Hap B that enhances nitrate uptake and confers high nitrogen use efficiency by increasing yield under low levels of nitrogen supply [5]. It was found that *OsNLP4* transactivates *OsNiR*-encoding nitrite reductase, which plays a key role in rice nitrogen assimilation. OsNLP4-OsNiR increases both the tiller number and yield by enhancing nitrogen assimilation [6]. Therefore, GWAS plays an important role in the study of low-nitrogen-tolerance-dominant genes in rice. Mining low-nitrogen-tolerant-dominant genes and using the GWAS method to study the functions of different genes under low levels of nitrogen are important contributions to the revelation of the complex molecular mechanism of low-nitrogen-tolerance traits in rice [7,8,9,10,11].

Research on the molecular mechanism of genes affecting low nitrogen tolerance can provide a theoretical basis for ascertaining the tolerance of rice to low levels of nitrogen. Based on the analysis of the genetic regulatory network, low-nitrogen-tolerance traits of rice are found to be controlled by multi-level molecular mechanisms. QTL mapping provides key information for analyzing the genetic basis of low-nitrogen-tolerance traits. Loss-of-function *ARE1* mutations delay senescence and increase grain yield by 10–20% under nitrogen limitation. *ARE1* modulates glutamate synthase complex assembly in chloroplasts, regulating nitrogen assimilation and source–sink dynamics [12]. The *OsNPF* gene family encodes nitrate transporters, which are widely involved in root nitrogen uptake, nitrogen redistribution and reuse, and low nitrogen signal regulation. *OsNPF7.6* can increase nitrate uptake in rice, thereby improving rice yield per plant and nitrogen-use efficiency [13]. *OsNPF6.5* (*NRT1.1B*) is specifically expressed in roots and is responsible for nitrate (NO_3_^−^) uptake. Under low-nitrogen conditions, the expression of some genes such as *OsNPF6.5* was up-regulated, which enhanced the ability of roots to capture low-nitrate concentrations in soil [14]. *OsNPF2.2* is involved in nitrogen transport in the xylem or phloem, transporting nitrogen absorbed by roots to shoots to ensure nitrogen supply to key organs [15]. *OsAMT1;1*, *OsAMT1;2,* and *OsAMT1;3* co-regulated the ammonium salt uptake of rice under low-nitrogen conditions, and the influx of high-affinity ammonium was regulated in the roots of rice seedlings through the differential expression of three members of the *OsAMT1* family under the conditions of changes in the nitrogen source and amount of daily light [16,17]. *OsAMT1.1* contributed significantly to NH_4_^+^ uptake under both low- and high-NH_4_^+^ conditions; it had the potential to improve nitrogen-use efficiency, plant growth, and grain yield; and it played an important role in the nitrogen and potassium homeostasis of rice [18,19]. Overexpressing the highly active transcript *OsGS1; 1b* can enhance nitrogen absorption and assimilation and improve nitrogen utilization efficiency [3]. Among them, the yields of materials with excellent alleles of *NRT1.1B* [14], *AMT1.1* [18] and *OsGS1;1* [3] were significantly higher than those of their controls, respectively, under low-nitrogen conditions. In addition, many QTLs have been reported in studies, which provide more experience in analyzing low nitrogen tolerance in rice [3,20,21].

Although some genes related to low nitrogen tolerance in rice have been cloned, it remains critically important to clone more low nitrogen tolerance genes and apply them to breeding. In this study, we performed a GWAS to detect QTL related to low nitrogen tolerance using the 562 multiparent advanced generation intercross (MAGIC) line population developed by eight *xian* parents. We further narrowed down the candidate QTL by integrating the results of GWAS using 284 rice resequencing germplasm resources. We performed annotation analysis on single nucleotide polymorphism (SNP) within the candidate region, which was followed by haplotype and bioinformatics analysis on the candidate genes within the candidate region affecting the low nitrogen tolerance-related traits. Finally, the candidate genes and their superior haplotypes affecting the traits related to low nitrogen tolerance were identified. Our results will enhance the knowledge of the genetic basis of tolerance to low nitrogen and provide valuable information for improving the tolerance to low nitrogen in rice-breeding programs.

## 2. Results

### 2.1. Phenotypic Variation

**Figure 1:**
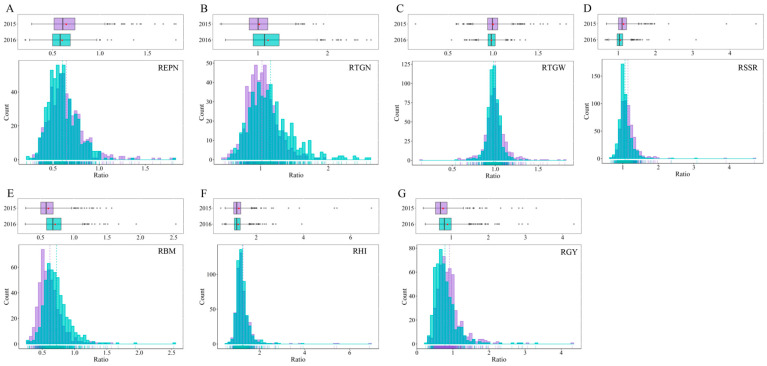
The phenotypic distribution of seven traits related to low nitrogen tolerance. The seven traits (**A**) REPN, (**B**) RTGN, (**C**) RTGW, (**D**) RSSR, (**E**) RBM, (**F**) RHI and (**G**) RGY indicated the ratio of effective panicle number per plant (EPN), total number of grains per panicle (TGN), thousand grains weight (TGW, g), seed setting rate (SSR, %), biomass (BM, g), harvest index (HI), and grain yield per plant (GY, g) of low to normal nitrogen conditions in this study.

The rice panels used in this study showed wide variations for all the measured traits with most traits following a normal distribution (Figure 1). Phenotypic data with broad variations are suitable for genome-wide association analysis to screen candidate genes affecting low-nitrogen tolerance-related traits. The traits REPN, RTGW and RHI demonstrated relatively high heritability values of 0.82, 0.79 and 0.81, respectively (Table S1), indicating minimal environmental influence on these traits. In contrast, RTGN and RSSR showed lower heritability values of 0.21 and 0.32, respectively (Table S1), indicating that these traits were greatly affected by the environment. The traits with high heritability are more likely to be mapped to the same QTL across different environments.

**Figure 2:**
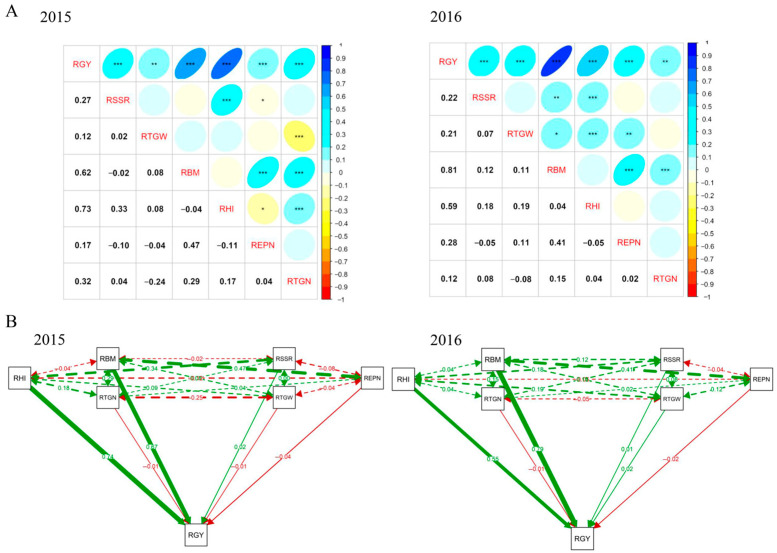
Correlation and path analysis among seven traits related to low nitrogen tolerance in 2015 and 2016. (**A**) Correlations among seven evaluated traits in 2015 and 2016. The areas and colors of ellipses (upper triangular) indicate the absolute value of corresponding *r* (lower triangular). Right and left oblique ellipses indicate positive and negative correlations, respectively. *, **, and *** represent significant correlations at *p* < 0.05, *p* < 0.01, and *p* < 0.001, respectively; (**B**) correlations among seven evaluated traits in 2015 and 2016. Full lines represent the direct effect of each trait on yield, whereas dotted lines represent the indirect effect; green lines represent the positive effect while red lines represent the negative effect. The size of the lines represents the path coefficients.

The pairwise phenotypic correlations among the measured traits were similar in 2015 and 2016 (Figure 2A). As expected, RGY showed significant positive correlations with all of the other six traits (RSSR, RTGW, RBM, RHI, REPN and RTGN) in both years. The strongest correlation coefficients were observed between RGY and RHI (*r* = 0.73) in 2015 and between RGY and RBM (*r* = 0.81) in 2016 (Figure 2A). RSSR showed significant positive correlations with RHI in both years. The RBM showed significant positive correlations with REPN and RTGN in both years (Figure 2A).

Different traits showed different contribution rates to the response of yield tolerance to low nitrogen through the path analysis of these yield components on the yield per plant (Figure 2B). Among them, RHI and RBM exhibited the greatest direct positive contributions to RGY in both years, whereas the other four traits (RTGN, RSSR, RTGW and REPN) exhibited no direct contributions to RGY (Figure 2B). The RSSR indirectly enhanced RGY through RHI, while REPN and RTGN mainly affected RGY via RBM (Figure 2B).

### 2.2. Genotype Analysis of the MAGIC Population in This Study

**Figure 3:**
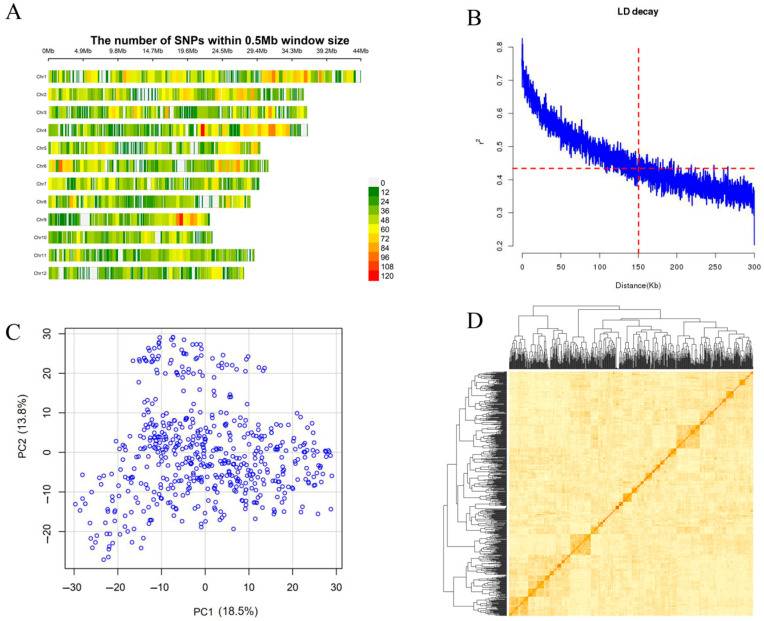
Genotype analysis of MAGIC populations used in this study. (**A**) The density distribution of SNPs on 12 chromosomes; (**B**) the linkage disequilibrium (LD) decay analyses in the MAGIC populations; (**C**) principal component analysis (PCA) plots for the first two components; (**D**) kinship (K) heat map of the MAGIC population.

When SNPs with missing rates over 20% and minor allele frequency (MAF) less than 5% were removed, a total of 27,958 high-quality SNPs remained for genotypic analysis. The number of markers per chromosome ranged from 1681 on chromosome 12 to 3870 on chromosome 1. The sizes of chromosome varied from 22.69 Mb (chromosome 9) to 43.95 Mb (chromosome 1) with a total genome size of 374.82 Mb. The average marker spacing was 13.14 kb, ranging from 11.36 kb (chromosome 1) to 16.37 kb (chromosome 12) (Figure 3A). The preliminary chromosomal region of each QTL was determined within 150 kb upstream and downstream of the lead SNP based on the linkage disequilibrium (LD) decay (Figure 3B), which was consistent with the previously reported linkage disequilibrium (LD) decay in 3 K RG [22].

There was no obvious clustering in the tested materials based on the principal component analysis (PCA) and kinship (K) analysis, suggesting that there was only one group or no obvious population structure in the MAGIC population (Figure 3C,D), which was likely because the parents used to create the MAGIC population originated from the *xian* subpopulation, and the alleles from different parents experienced free recombination during the construction of the MAGIC population [23].

### 2.3. Genome-Wide Association Identifies Significant QTLs Using MAGIC Population

A total of 32 different QTLs for all traits were identified in 2015 and 2016, ranging from one QTL for RTGW to nine QTLs for RHI and REPN (Table 1 and Figure S1). Among them, 15 and 19 QTL were detected in 2015 and 2016, respectively, and two QTLs were detected in both years (Table 1 and Figure S1).

For RGY, four QTLs were mapped on chromosomes 1, 6, 9 and 11. Among them, *qRGY1* and *qRGY9* were only detected in 2016, accounting for 3.25% and 3.47% of phenotypic variance, respectively. The *qRGY11* was only detected in 2015, explaining 6.77% of phenotypic variance. The *qRGY6* was detected in both years with an average phenotypic variance explanation of 6.18% (Table 1 and Figure S1).

Only two QTLs (*qRBM6.1* and *qRBM6.2*) were detected for RBM, and both were located on chromosome 6. The *qRBM6.1* and *qRBM6.2* were detected in 2016 and 2015, accounting for 1.95% and 1.98% of the phenotypic variance, respectively (Table 1 and Figure S1).

For RHI, a total of nine QTLs were detected on chromosomes 2, 3, 4, 5, 6, 7 and 11. Five QTLs *(qRHI2.1*, *qRHI2.2*, *qRHI5.1*, *qRHI7* and *qRHI11*) detected in 2015 explained 3.45%, 3.31%, 4.01%, 4.27% and 7.74% of the phenotypic variance, respectively. The *qRHI3*, *qRHI4* and *qRHI5.2* were detected in 2016, accounting for 3.10%, 3.09% and 4.02% of the phenotypic variance, respectively (Table 1 and Figure S1).

For REPN, nine QTLs were detected on chromosomes 3, 6, 7, and 8. Seven QTLs (*qREPN3, qREPN6, qREPN7.1, qREPN7.2, qREPN7.3, qREPN7.4* and *qREPN7.5*) were detected in 2016, explaining phenotypic variances ranging from 2.69% to 2.84%. The remaining two QTLs (*qREPN7.2* and *qREPN8*), detected in 2015, accounted for 3.40% and 3.05% of the variance, respectively (Table 1 and Figure S1).

A total of three (*qRTGN2, qRTGN3* and *qRTGN6*), four (*qRSF4, qRSF10, qRSF11* and *qRSF12*) and one (*qRTGW6*) QTL were detected for RGTN, RSF and RTGW, respectively (Table 1 and Figure S1).

Among the 32 QTLs detected for the low nitrogen tolerance-related traits in rice using the MAGIC population, the four QTLs, including *qRGY6*, *qRBM6.2*, *qRHI6* and *qRTGN6,* were co-located within the region of 2.78–3.11 Mb on chromosome 6. Another QTL cluster, including *qRGY11* and *qRHI11*, influencing both RGY and RHI, was mapped to the region of 9.09–9.39 Mb on chromosome 11 (Table 1 and Figure S1). These two QTL clusters, affecting multiple traits, were the important candidate regions to search the candidate genes related to low nitrogen tolerance.

### 2.4. Important QTLs Were Further Identified Combined with the Result of Genome-Wide Association Analysis Using Germplasm Accessions

**Figure 4:**
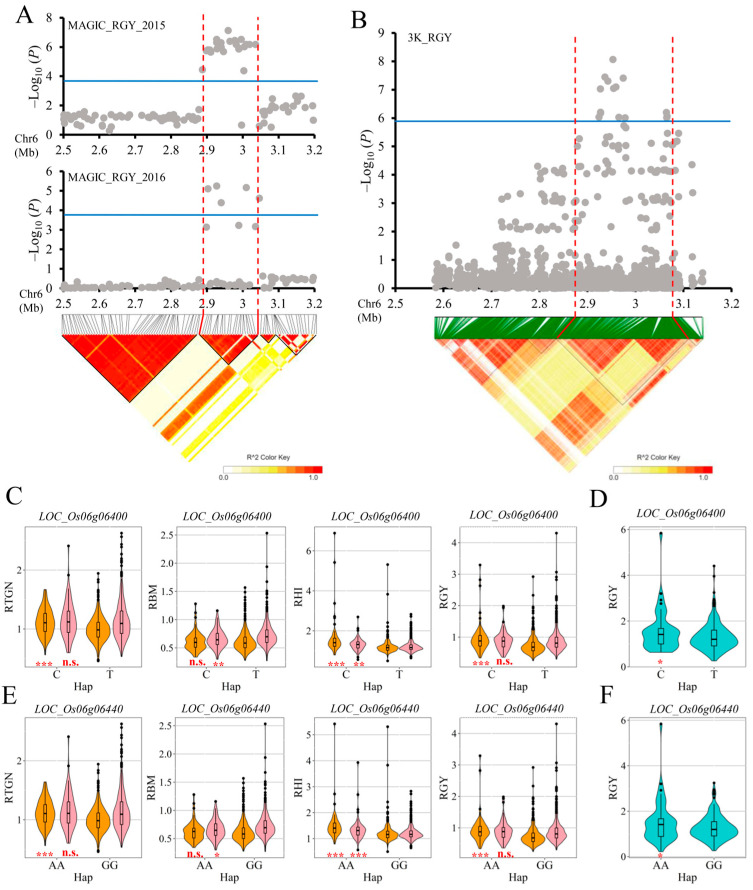
Candidate genes analysis of the QTL cluster harboring *qRGY6*, *qRBM6.2*, *qRHI6* and *qRTGN6* on chromosome 6. Local Manhattan plot (top) and LD block (bottom) surrounding the peak SNP on chromosome 6 in (**A**) MAGIC population and (**B**) germplasm resources. Blue lines show the threshold to determine significant SNPs. Red lines indicate the candidate region for the peak SNP. Haplotype analysis of *LOC_Os06g06400* in (**C**) MAGIC population and (**D**) germplasm resources. Haplotype analysis of *LOC_Os06g06440* in (**E**) MAGIC population and (**F**) germplasm resources. Orange, pink and cyan indicated 2015, 2016 and 2022. Asterisks represent significant difference determined by Student’s t test at *** *p* < 0.001, ** *p* < 0.01, * *p* < 0.05, and not significant (n.s.).

In order to further validate the candidate QTLs affecting low-nitrogen-tolerance-related traits, 284 resequenced germplasm varieties resources from 3K-RGP including 147 *geng* accessions and 137 *xian* accessions were planted in the field trial under low and normal nitrogen conditions in Shenyang (41.48° N, 123.34° E) of Liaoning province in 2022. We measured the ratio of grain yield per plant (RGY) of the low nitrogen condition to that of the normal condition, and they all showed wide variations (Figure S2). After quality control of genotypes (GENO < 20% and MAF > 5%), 2,803,682 high-quality SNPs were retained for population structure and genome-wide association analysis. A principal component analysis (PCA) and the kinship matrix (K) were performed to examine the population structure, and they were used in the following association analysis (Figure S3A,B). Based on the effective number of independent markers, the suggestive *P*-value threshold of association (1/N) was 2.53 × 10^−6^ for germplasm varieties, which was used to claim significant SNP–trait associations (Figure S3C and Figure 4B). According to the result of genome-wide association analysis using germplasm varieties, there were 15 SNPs significantly associated with low nitrogen tolerance located in the region 2.78–3.11 Mb on chromosome 6, which was consistent with mapping results using the MAGIC population (Figure S3C,D; Table 1). However, no QTL affecting RGY was mapped on chromosome 11 in the germplasm panel (Figure S3C). Therefore, the QTL cluster, including *qRGY6*, *qRBM6.2*, *qRHI6* and *qRTGN6*, was regarded as the most likely candidate region affecting rice tolerance to low nitrogen, and the candidate genes would be mined in this region.

The candidate region of *qRGY6* was predicted to span from 2.898 Mb to 3.046 Mb (148 kb) on chromosome 6, which overlapped with the QTL mapped by GWAS using the germplasm varieties, ranging from 2.874 Mb to 3.074 Mb (200 kb) based on the LD block analysis (Figure 4A,B). Thus, the region from 2.898 Mb to 3.046 Mb, containing 21 genes (Table S2) according to the China rice data center (https://www.ricedata.cn/gene/, accessed on 25 March 2025), was identified as the final candidate region associated with tolerance to low nitrogen. Among the total 20 SNPs within the region used in this study, three, seven, one, seven and two SNPs were located in the promoter, 5′-UTR, intron, CDS (missense variant) and 3′-UTR regions of 13 of the 21 genes, respectively. No SNPs were detected on the other eight genes (*LOC_Os06g06310, LOC_Os06g06340, LOC_Os06g06350, LOC_Os06g06370, LOC_Os06g06410, LOC_Os06g06430, LOC_Os06g06460* and *LOC_Os06g06480*) (Table S2). We performed haplotype analysis using these 20 SNPs for the 13 candidate genes based on the four low-nitrogen-tolerance-related traits, including RGY, RBM, RHI and RTGN. Except for *LOC_Os06g06470*, the other 12 candidate genes all showed significant differences among different haplotypes of RGY_15, RHI_15, RTGN_15, RBM_16 and RHI_16 (Table S3). Among the 12 candidate genes, only four genes (*LOC_Os06g06300*, *LOC_Os06g06330*, *LOC_Os06g06400* and *LOC_Os06g06440*) contained missense mutations in the CDS region and exhibited significant differences among different haplotypes in the MAGIC populations (Table S3; Figure 4). We furtherly performed haplotype analysis of these four genes using the RGY data from the 284 resequenced germplasm resources, and there were significant differences among haplotypes for *LOC_Os06g06400* and *LOC_Os06g06440* (Table S4; Figure 4D,F). For *LOC_Os06g06400*, when the haplotype changed from C to T at rs6_2988724, the average phenotypic values for RGY with haplotype C were significantly higher than those with haplotype T at the significance levels of 0.001 and 0.05 in MAGIC populations and resequenced germplasm resources, respectively (Figure 4C,D). Similarly, for *LOC_Os06g06440*, the average RGY values with haplotype AA were significantly higher than those with haplotype GG at the significance level 0.001 and 0.05 in MAGIC populations and resequenced germplasm resources, respectively (Figure 4E,F).

The *LOC_Os06g06400* was predicted to encode the NBS-LRR type disease resistance protein (https://www.ricedata.cn/gene/, accessed on 25 March 2025), but its role in abiotic stress response remains unreported. The *LOC_Os06g06440* encodes the ATP-Binding Cassette (ABC) transporter, a family with reported roles in abiotic stress responses [24,25,26,27,28]. Thus, *LOC_Os06g06440* was the most likely candidate gene affecting low-nitrogen-tolerance-related traits in rice within the region from 2.898 Mb to 3.046 Mb (148 kb) on chromosome 6.

### 2.5. Candidate Genes Analysis

**Figure 5:**
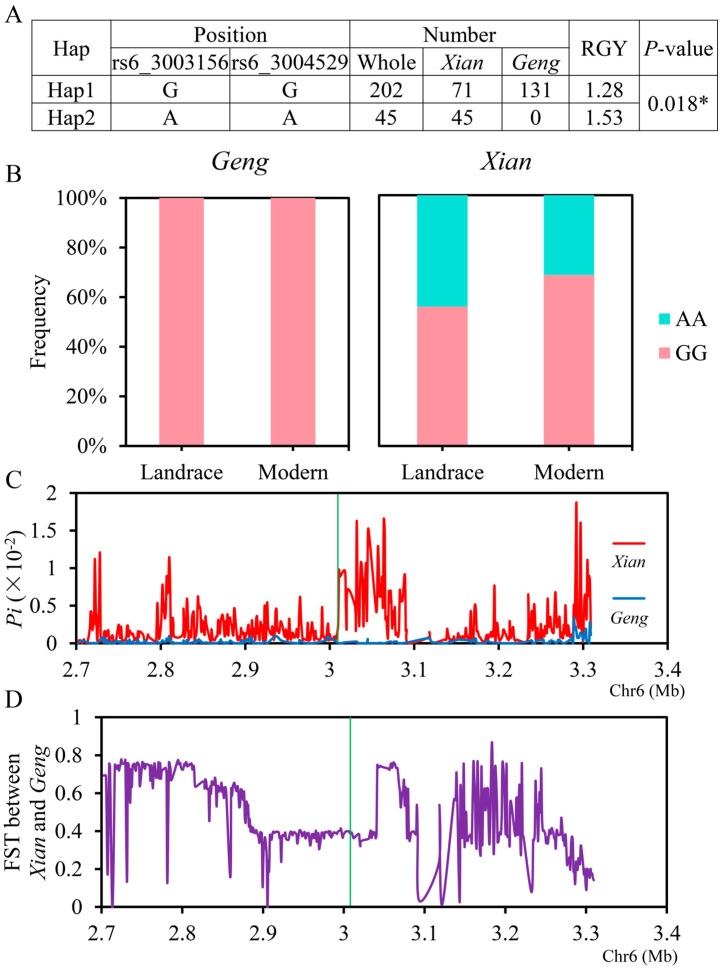
Haplotype analyses for the RGY and nucleotide diversity of the *LOC_Os06g06440* candidate gene at *qRGY6*. (**A**) Haplotype analyses of the mean RGY in 284 rice germplasm resources. * represents significant correlations at *P* < 0.05. (**B**) Frequencies of different haplotypes of *LOC_Os06g06440* in *Xian* and *Geng* landraces and modern varieties. (**C**) Nucleotide diversity (*Pi*) and (**D**) the fixation index (FST) between *Xian* and *Geng* subpopulations for the 600 kb genomic region flanking *LOC_Os06g06440*. The green line indicates the location of *LOC_Os06g06440*.

Significant differences in RGY were observed between Hap1 (GG) and Hap 2 (AA) of *LOC_Os06g06440* (Figure 5A). The frequencies of both haplotypes were significantly associated with the rice subgroups according to Fisher’s exact tests (Table S5). Additionally, all accessions with the high-RGY haplotypes Hap2 (*n* = 45) belonged to the *xian* subgroup, suggesting that the superior haplotype of the *LOC_Os06g06440* existed in *xian* subgroup. In contrast, 35.1% and 64.9% of the accessions with the low-RGY haplotypes Hap1 (*n* = 202) belonged to the *xian* and *geng* subgroup, respectively (Figure 5A). Moreover, within the *xian* subgroup, the frequency of Hap1 increased from 55.6% in landrace (LAN) to 68.1% in modern varieties (MV) (Figure 5B). We analyzed the nucleotide diversity (*Pi*) and fixation index statistics (FST) for a 600 kb region flanking *LOC_Os06g06440* in the *xian* and *geng* subgroups (Figure 5C, D). The *Pi* of *xian* subgroup was significantly higher than that of *geng* subgroup for the *LOC_Os06g06440* region (Figure 5C), suggesting stronger selective pressure in *xian* varieties. In terms of fixation index statistics (FST > 0.25), *LOC_Os06g06440* had obvious differentiating characteristics between *xian* and *geng* subgroups (Figure 5D). Then, we chose nine *xian* varieties (IRIS_313-11624, IRIS_313-10932, CX382, B067, B249, IRIS_313-8215, IRIS_313-10544, B052, and B233) carrying superior haplotype (AA) and exhibiting higher RGY (>2) for the molecular marker-assisted selection of low nitrogen tolerance in rice (Table S6).

## 3. Discussion

The MAGIC population used in this study showed broad variations for all the measured traits (Figure 1), suggesting that the phenotypic data with broad variations could be used for genome-wide association analysis to identify candidate genes associated with low-nitrogen-tolerance-related traits in this study [29].

Based on the correlation analysis result among different traits, RGY showed significantly positive correlations with all of the other six traits (RSSR, RTGW, RBM, RHI, REPN and RTGN), suggesting that all the six traits could affect the RGY, which was similar with the result of path analysis. The RSSR showed significantly positive correlations with RHI, and the RBM showed significantly positive correlations with REPN and RTGN (Figure 2A). These observations were consistent with the results of path analysis, which indicated that the RSSR indirectly and positively affected RGY through RHI, and the REPN and RTGN mainly indirectly and positively affected RGY through RBM. These results suggested that the RBM and RHI were directly and closely related to RGY, whereas RSSR, REPN and RTGN indirectly affected RGY by modulating RBM or RHI. The relationship among different yield components under low-nitrogen conditions in this study provided a new approach for identifying yield-related phenotypes with low nitrogen tolerance.

The environmental factors, such as temperature and precipitation, varied between years and could influence quantitative traits, leading to different mapping results through GWAS in different years. The QTL jointly mapped between different years or different methods were relatively reliable for identifying candidate genes, and this approach was widely adopted in GWAS. In this study, we first performed genome-wide association analysis using the MAGIC population to identify QTL that affected low nitrogen tolerance. Then, we used germplasm resources for genome-wide association to further determine the QTLs identified by MAGIC populations. The candidate region from 2.898 Mb to 3.046 Mb on chromosome 6, affecting the tolerance to low nitrogen, were mapped in both MAGIC populations and germplasm resources. The mutual verification of the mapping results of different groups could reduce the false positives of GWAS and enhance the accuracy of QTL mapping results. Therefore, the QTL cluster modulating *qRGY6*, *qRBM6.2*, *qRHI6* and *qRTGN6,* mapped in 2.898–3.046 Mb on chromosome 6, was a reliability candidate region affecting the tolerance to low nitrogen. This candidate region was a unreported QTL for low nitrogen tolerance. It could be used as a QTL for identifying new genes resistant to low nitrogen.

Of the total 20 SNPs within the candidate region used in the MAGIC populations, seven SNPs were located on the missense variant in CDS regions of four genes, including *LOC_Os06g06300*, *LOC_Os06g06330*, *LOC_Os06g06400* and *LOC_Os06g06440*. Significant differences were observed among different haplotypes of these four genes in MAGIC populations. However, significant differences among haplotypes were only detected in the gene *LOC_Os06g06400* and *LOC_Os06g06440* in germplasm resources. Thus, we considered that the *LOC_Os06g06400* and *LOC_Os06g06440* were the important candidate genes affecting the tolerance to low nitrogen.

The *LOC_Os06g400* was predicted to encode an NBS-LRR type disease resistance protein (https://www.ricedata.cn/gene/, accessed on 10 April 2025). The *NBS-LRR* (Nucleotide-Binding Site-Leucine-Rich Repeat) gene family in rice was an important type of disease resistance (R) gene, whose main function was to participate in the immune defense of plants against pathogens (such as bacteria, fungi, viruses and nematodes) [30]. However, no previous studies have reported its function in response to abiotic stress. The *LOC_Os06g440* could encode the ATP-Binding Cassette (ABC) transporter, and some genes belonged to ABC trans-porter family were reported on the function in response to abiotic stress [24,25,26,27,28]. For example, *OsABCB25* (*LOC_Os03g54790*) and *OsABCI12* (*LOC_Os06g48060*) could regulate the tolerance of rice to aluminum poisoning [24,25]. *OsABCB23* (*LOC_Os06g03770*), *OsABCI8* (*LOC_Os11g29850*) and *OsABCB14* (*LOC_Os04g38570*) were involved in the dynamic balance of iron ions in rice to maintain their normal growth [26,27,28]. Thus, *LOC_Os06g06440* was the most likely gene affecting low-nitrogen-tolerance-related traits in rice within the region from 2.898 Mb to 3.046 Mb (148 kb) on chromosome 6.

The average phenotypic values for RGY with haplotype AA of *LOC_Os06g06440* was significantly higher than those with haplotype GG at the significance levels 0.001 and 0.05 in MAGIC populations and resequenced germplasm resources, respectively. Whether these two non-synonymous mutations were functional sites of the *LOC_Os06g06440* still required further validation through the creation of transgenic materials. The frequencies of both two haplotypes were significantly associated with the rice subgroups according to Fisher’s exact tests, *Pi* and FST analysis. Additionally, all the accessions with the high-RGY haplotypes Hap2 belonged to the *xian* subgroup. These results suggested that *LOC_Os06g06440* had obvious differentiation characteristics between the *xian* and *geng* subgroups, and the superior haplotype of the *LOC_Os06g06440* existed in the *xian* subgroup. Finally, we chose nine *xian* varieties (IRIS_313-11624, IRIS_313-10932, CX382, B067, B249, IRIS_313-8215, IRIS_313-10544, B052, and B233) carrying superior haplotype (AA) and displaying higher RGY (>2) for the molecular marker-assisted selection of low nitrogen tolerance in rice. Further research is needed to understand the regulatory mechanism of *LOC_Os06g06440* that contributes to low nitrogen tolerance in rice after gene cloning.

## 4. Materials and Methods

### 4.1. Plant Materials

Eight elite parents (Table S7) were selected based on genetic diversity to develop a multiparent advanced generation intercross (MAGIC) population at the IRRI since 2008 [31]. The eight-parental MAGIC population, including 562 lines, was used as materials for investigating low-nitrogen-tolerance-related traits. In addition, 284 resequenced germplasm varieties with similar heading dates from the 3K RGP were selected for this study [32]. These varieties, including 137 *xian* and 147 *geng* varieties, had a rich phenotype with globally diverse genetic backgrounds (Table S6).

### 4.2. Field Trial and Phenotypic Investigation

The 562 lines from the eight- parental MAGIC population were planted in Shenzhen (22.6° E, 114.07° N) of Guangdong province in 2015 and 2016 under low nitrogen (LN) and normal nitrogen (NN) conditions. The LN field was created by planting rice crops in the field with zero nitrogen application (but normal application of P and K fertilizers) for 10 consecutive seasons in the past 5 years. In the normal (non-stress) field, 140 kg N ha^−1^ was applied (∼70% used as basal and 30% applied in 15 days after transplanting). Phosphorus (40 kg ha^−1^) and potassium (40 kg ha^−1^) fertilizers were also applied as basal under stress and normal conditions. The paddy soil in the LN field before the experiment had a pH of 6.18, organic matter of 4.32 g kg^−1^, total N of 310 mg kg^−1^, available P of 79.2 mg kg^−1^, and available K of 165 mg kg^−1^. Contrarily, the paddy soil in the normal field before the experiment had a pH of 5.97, organic matter of 10.9 g kg^−1^, total N of 920 mg kg^−1^, available P of 88.8 mg kg^−1^, and available K of 155 mg kg^−1^. And the 284 resequenced germplasm varieties from 3K-RGP were planted in pots, and the pot experiment was conducted in the field at Shenyang (41.48° N, 123.34° E) of Liaoning province in 2022 under low nitrogen (LN) conditions and normal nitrogen (NN) conditions, which contained similar N contents to the planting conditions of the MAGIC populations [32,33]. The tested materials were all planted in a randomized complete block design with three replications. Each accession was planted in five rows with eight individuals in each row at a spacing of 25 cm between rows and 17 cm between plants.

At maturity, eight uniform plants in the middle of each plot were bulk-harvested and air-dried for three months to constant weight in the drying houses. The seven yield-related traits were measured for MAGIC populations, including the effective spike number per plant (EPN), total number of grains per panicle (TGN), seed setting rate (SSR, %), thousand grains weight (TGW, g), biomass (BM, g), harvest index (HI), and grain yield per plant (GY, g), in low nitrogen and normal nitrogen conditions, respectively. The grain yield per plant (GY, g) was measured under both low nitrogen and normal nitrogen conditions for the 284 germplasm varieties in 2022. We utilized the ratio of trait values measured under low-nitrogen conditions to those measured under normal conditions as the trait index for evaluating low-nitrogen tolerance, including REPN, RTGN, RSSR, RTGW, RBM, RHI, and RGY.

### 4.3. Genotyping Data Analysis

Genotyping of the MAGIC populations was reported by Meng et al. [31]. The raw genotype data of the 284 accessions were obtained from the 3K RGP Rice SNP-Seek Database website (https://snp-seek.irri.org/, accessed on 18 December 2024). Those SNPs with missing rates over 20% and minor allele frequency (MAF) less than 5% were removed using PLINK 1.9 software [34]. Finally, further analysis was performed using the retained high-quality 27,958 and 2,803,682 SNPs for the MAGIC population and 3K RGP panel, respectively. The effective number of independent markers (N) was calculated using the GEC software [35], and the suggestive *p*-value thresholds of association (1/N) were 1.45 × 10^−4^ and 2.53 × 10^−6^ for the MAGIC populations and germplasm varieties, respectively, which were used to claim significant SNP–trait associations. Principal component analysis (PCA) and kinship matrix (K) were performed by PLINK 1.9 software [34] to examine the population structure and used in the following association analysis.

### 4.4. Genome-Wide Association Study (GWAS) of QTL

We performed a GWAS to identify SNPs significantly associated with seven measured traits (REPN, RTGN, RSSR, RTGW, RBM, RHI, and RGY) using the 27,958 SNPs and the mean trait values of the 562 MAGIC accessions using PLINK 1.9 software [34]. Then, we performed a GWAS to identify SNPs significantly associated with RGY using the 2,803,682 SNPs and the mean trait values of the 284 germplasm varieties using PLINK 1.9 software. In this study, the mixed linear model (MLM) PCA + K was used in the association analysis. Manhattan plots were plotted by the R package “CMplot” using the GWAS results (https://github.com/YinLiLin/CMplot, accessed on 5 February 2025). The preliminary chromosomal region of each QTL was determined within 150 kb upstream and downstream of the lead SNP based on the linkage disequilibrium (LD) decay (Figure 3B).

### 4.5. Candidate Gene Identification for the Important QTL

We selected important QTLs which were simultaneously identified in both years for one trait or affected more than one trait in one year to identify candidate genes affecting target traits based on the GWAS results for the low-nitrogen-tolerance-related traits in the MAGIC populations. The LD block region where the significant trait-associated SNPs were situated was defined as the candidate region. The LDs between SNPs were evaluated using squared Pearson’s correlation coefficient (*r*^2^) calculated with the R package “genetics”. LD heatmaps surrounding peaks in the GWAS were constructed with the R package “LD heatmap” [36]. Candidate regions were estimated using an *r*^2^ ≥ 0.6 [37]. The genes where the SNPs used in this study were located were identified as the candidate genes for each important QTL based on the Rice Annotation Project Database [38]. Then, we further determined the accuracy of candidate intervals based on the results of GWAS of germplasm resources.

Among these important QTLs, genes containing the available SNPs were selected as important candidate genes affecting LN tolerance-related traits. And then, we performed haplotype analysis using 284 accessions selected from the 3K [32] for each of the candidate genes using all non-synonymous SNPs. Finally, the most likely candidate genes were selected for comprehensive analysis based on the significance of haplotype analyses (analysis of variance (ANOVA)) and their functional annotations. Two-sided Fisher’s exact tests in R were used to compare haplotype frequencies between the *xian* and *geng* subgroups. The nucleotide diversity (*Pi*) and FST for each 1 kb window across the genome, with an overlapping 5 kb step size, were calculated for the 600 kb region flanking the candidate genes with the Variscan program (version 2.0.3) [39].

### 4.6. Statistical Analysis of Data

Differences in the phenotypic values between different nitrogen conditions were examined by Student’s *t* test. Differences in the phenotypic values between the haplotypes of candidate genes were examined by a one-way ANOVA, including Student’s *t* test and Duncan’s multiple range test. Duncan’s multiple range test was conducted to determine the significance of any differences (*p* < 0.05) when the number of groups is more than two. Student’s *t* test (two-tailed) was used to determine exact *p*-values when the number of groups is two. The heritability (*H*) for each trait was calculated by *Vg*/(*Vg* + *Vge/L* + Ve/(L*R)). The *Vg* represented epigenetic variance, *Vge* represented the gene–environment interaction variation, *L* represented the number of environments, *Ve* represented environmental variation, and *R* represented the number of repetitions. All the analyses were performed in R 4.3.2 software [36].

## 5. Conclusions

In this study, a total of 562 MAGIC line population and 284 germplasm varieties were used for genome-wide association analysis (GWAS) and haplotype analysis, aiming to detect quantitative trait loci (QTL) and candidate genes associated with low-nitrogen tolerance. The RBM and RHI were directly and closely related to RGY, while the RSSR indirectly and positively affected RGY through RHI, and the REPN and RTGN mainly indirectly and positively affected RGY through RBM based on the path analysis results. *LOC_Os06g06440* was the most likely candidate gene affecting RGY in rice within the region from 2.898 Mb to 3.046 Mb (148 kb) on chromosome 6. Nine *xian* varieties (IRIS_313-11624, IRIS_313-10932, CX382, B067, B249, IRIS_313-8215, IRIS_313-10544, B052, and B233) carrying superior haplotype (AA) of *LOC_Os06g06440* and exhibiting higher RGY were recommended for the molecular marker-assisted breeding of low nitrogen tolerance in rice. However, further research is needed to understand the regulatory mechanism of *LOC_Os06g06440* that contributes to low nitrogen tolerance in rice after gene cloning.

## Figures and Tables

**Table 1:** QTLs identified for low-nitrogen-tolerance-related traits by GWAS using MAGIC population.

Traits	QTL	Year	Peak SNP	Allele ^a^	QTL Region (Mb)	*p*-Value	*R^2^* (%) ^b^
RGY	*qRGY1*	2016	rs1_38797291	C/A	38.65–38.95	5.19 × 10^−5^	3.25
	*qRGY6*	2015	rs6_2959067	G/A	2.81–3.11	7.42 × 10^−8^	6.00
		2016	rs6_2926939	A/G	2.78–3.08	5.68 × 10^−6^	6.36
	*qRGY9*	2016	rs9_22349187	G/A	22.20–22.50	2.88 × 10^−5^	3.47
	*qRGY11*	2015	rs11_9235660	G/A	9.09–9.39	1.18 × 10^−8^	6.77
RBM	*qRBM6.1*	2016	rs6_2123411	C/T	1.97–2.27	3.72 × 10^−6^	1.95
	*qRBM6.2*	2015	rs6_2945886	A/G	2.80–3.10	3.17 × 10^−6^	1.98
RHI	*qRHI2.1*	2015	rs2_26804244	T/C	26.65–26.95	5.62 × 10^−5^	3.45
	*qRHI2.2*	2015	rs2_27601760	A/C	27.45–27.75	7.92 × 10^−5^	3.31
	*qRHI3*	2016	rs3_2221169	G/A	2.07–2.371	9.75 × 10^−5^	3.10
	*qRHI4*	2016	rs4_1056244	T/C	0.91–1.21	9.82 × 10^−5^	3.09
	*qRHI5.1*	2015	rs5_26492799	C/T	26.34–26.64	1.47 × 10^−5^	4.01
	*qRHI5.2*	2016	rs5_27940204	A/G	27.79–28.09	9.49 × 10^−6^	4.02
	*qRHI6*	2015	rs6_2959067	G/A	2.81–3.11	1.40 × 10^−9^	7.98
		2016	rs6_2959067	G/A	2.81–3.11	8.39 × 10^−6^	4.07
	*qRHI7*	2015	rs7_29638146	C/T	29.49–29.79	7.91 × 10^−6^	4.27
	*qRHI11*	2015	rs11_9235660	G/A	9.09–9.39	2.37 × 10^−9^	7.74
REPN	*qREPN3*	2016	rs3_3821913	T/C	3.67–3.97	9.91 × 10^−5^	2.77
	*qREPN6*	2016	rs6_397816	A/G	0.25–0.55	1.21 × 10^−4^	2.70
	*qREPN7.1*	2016	rs7_11632495	T/C	11.48–11.78	1.25 × 10^−4^	2.69
	*qREPN7.2*	2016	rs7_12808093	T/C	12.66–12.96	1.25 × 10^−4^	2.69
	*qREPN7.3*	2016	rs7_14490553	T/G	14.34–14.64	1.25 × 10^−4^	2.69
	*qREPN7.4*	2016	rs7_15026705	C/T	14.88–15.18	8.25 × 10^−5^	2.84
	*qREPN7.5*	2016	rs7_25930301	T/C	25.78–26.08	1.00 × 10^−4^	2.77
	*qREPN7.6*	2015	rs7_27630228	T/A	27.48–27.78	1.83 × 10^−5^	3.40
	*qREPN8*	2015	rs8_5126577	C/T	4.98–5.28	4.88 × 10^−5^	3.05
RTGN	*qRTGN2*	2016	rs2_24629837	A/G	24.48–24.78	1.41 × 10^−4^	2.72
	*qRTGN3*	2016	rs3_5899301	T/G	5.75–6.05	1.13 × 10^−4^	2.80
	*qRTGN6*	2015	rs6_2928178	G/A	2.78–3.08	5.87 × 10^−5^	3.09
RSF	*qRSF4*	2015	rs4_31547230	G/A	31.40–31.70	1.14 × 10^−10^	8.21
	*qRSF10*	2015	rs10_16807912	A/G	16.66–16.96	1.02 × 10^−4^	2.91
	*qRSF11*	2015	rs11_17412899	C/T	17.26–17.56	1.22 × 10^−4^	2.84
	*qRSF12*	2016	rs12_7593705	G/A	7.44–7.74	3.40 × 10^−6^	4.08
RTGW	*qRTGW6*	2016	rs6_2783620	A/G	2.63–2.93	1.43 × 10^−4^	1.89

^a^ Major/Minor allele. ^b^ Phenotypic variance explained by the peak SNP.

## Data Availability

The original contributions presented in the study are included in the article/Supplementary Material. Further inquiries can be directed to the corresponding authors.

## Supplementary Materials

The following supporting information can be downloaded at https://www.mdpi.com/article/10.3390/ijms26125749/s1.

## Abbreviations

The following abbreviations are used in this manuscript:

GWAS	genome-wide association study
LD	linkage disequilibrium
QTL	quantitative trait loci
MAGIC	multiparent advanced generation intercross
LN	low nitrogen conditions
NN	normal nitrogen conditions
EPN	effective spike number per plant
TGN	total number of grains per panicle
SSR	seed setting rate
TGW	thousand grains weight
BM	biomass
HI	harvest index
GY	grain yield per plant
MAF	minor allele frequency
PCA	principal component analysis
K	kinship matrix
MLM	mixed linear model
ANOVA	analysis of variance
