# Supplementary material for: Identification of Advantaged Genes for Low-Nitrogen-Tolerance-Related Traits in Rice Using a Genome-Wide Association Study
Source: Int J Mol Sci. 2025 Jun 16;26(12):5749. doi: 10.3390/ijms26125749 (PMC12193684; doi:10.3390/ijms26125749)
Supplement: Supplementary file 1 [file ijms-26-05749-s001.zip › figure S1.pptx]

## Slide 1
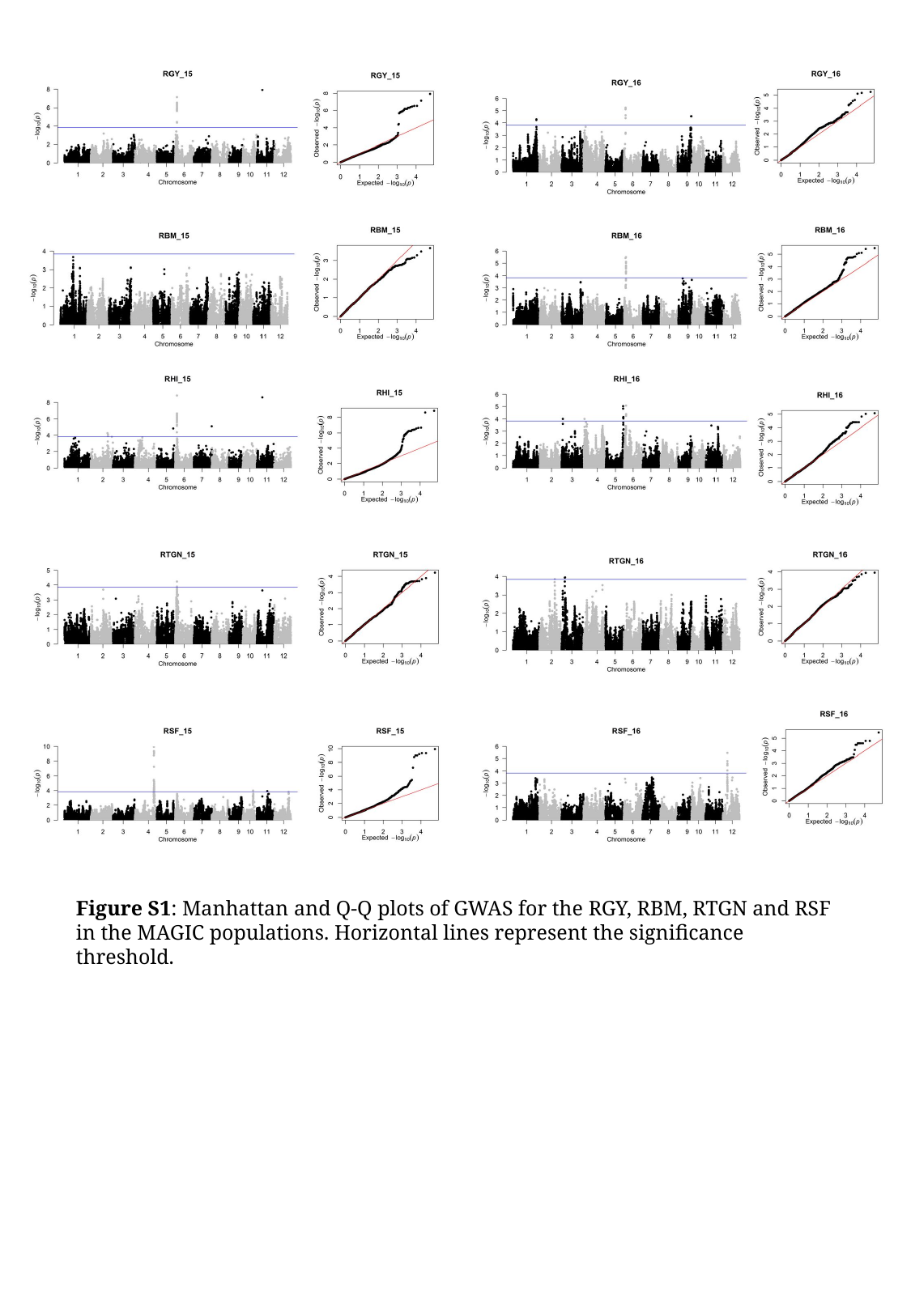

Figure S1: Manhattan and Q-Q plots of GWAS for the RGY, RBM, RTGN and RSF in the MAGIC populations. Horizontal lines represent the significance threshold.
