# Supplementary material for: Identification of Advantaged Genes for Low-Nitrogen-Tolerance-Related Traits in Rice Using a Genome-Wide Association Study
Source: Int J Mol Sci. 2025 Jun 16;26(12):5749. doi: 10.3390/ijms26125749 (PMC12193684; doi:10.3390/ijms26125749)
Supplement: Supplementary file 1 [file ijms-26-05749-s001.zip › figure S2.pptx]

## Slide 1
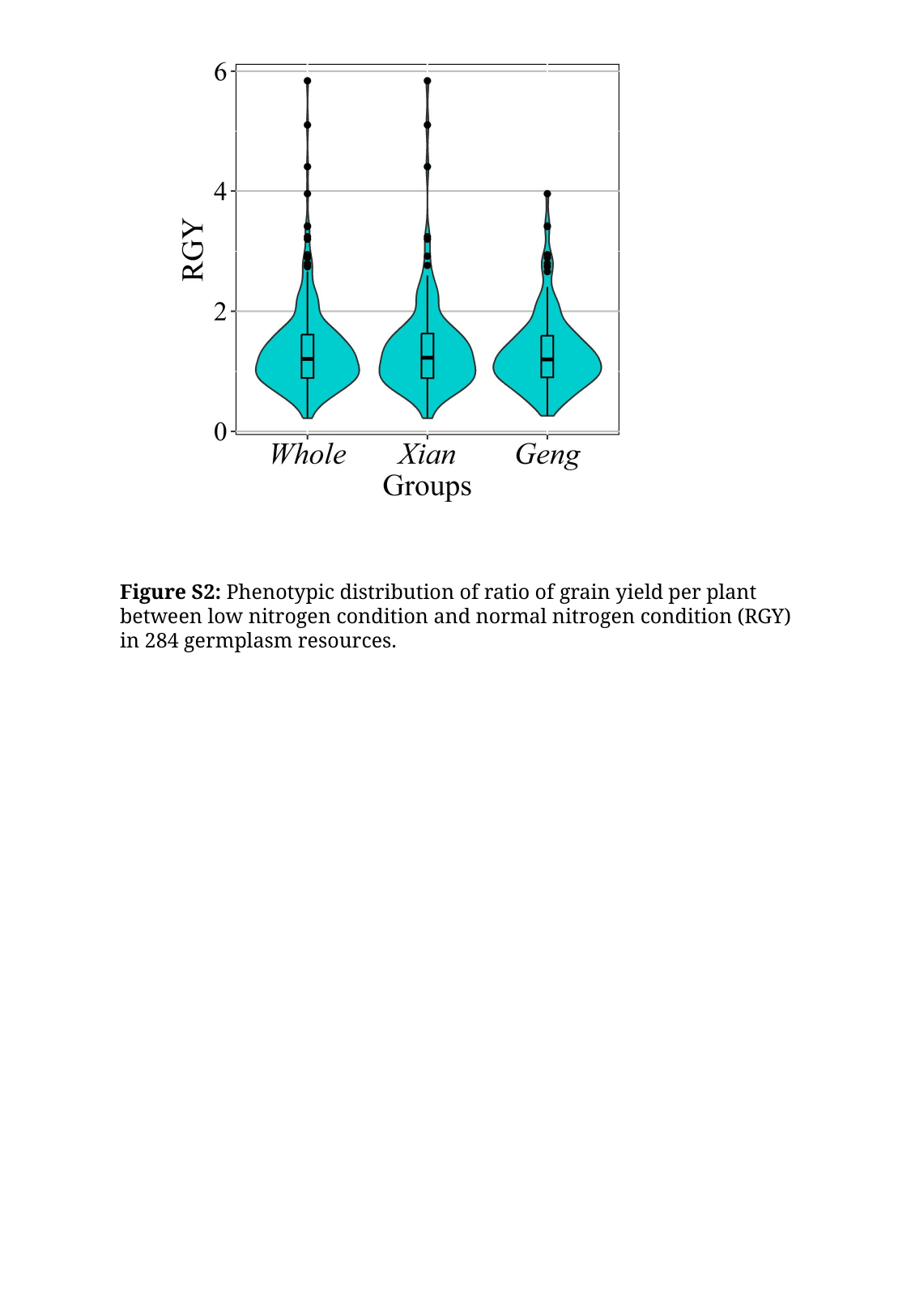

Figure S2: Phenotypic distribution of ratio of grain yield per plant between low nitrogen condition and normal nitrogen condition (RGY) in 284 germplasm resources.
