# Supplementary material for: Identification of Advantaged Genes for Low-Nitrogen-Tolerance-Related Traits in Rice Using a Genome-Wide Association Study
Source: Int J Mol Sci. 2025 Jun 16;26(12):5749. doi: 10.3390/ijms26125749 (PMC12193684; doi:10.3390/ijms26125749)
Supplement: Supplementary file 1 [file ijms-26-05749-s001.zip › figure S3.pptx]

## Slide 1
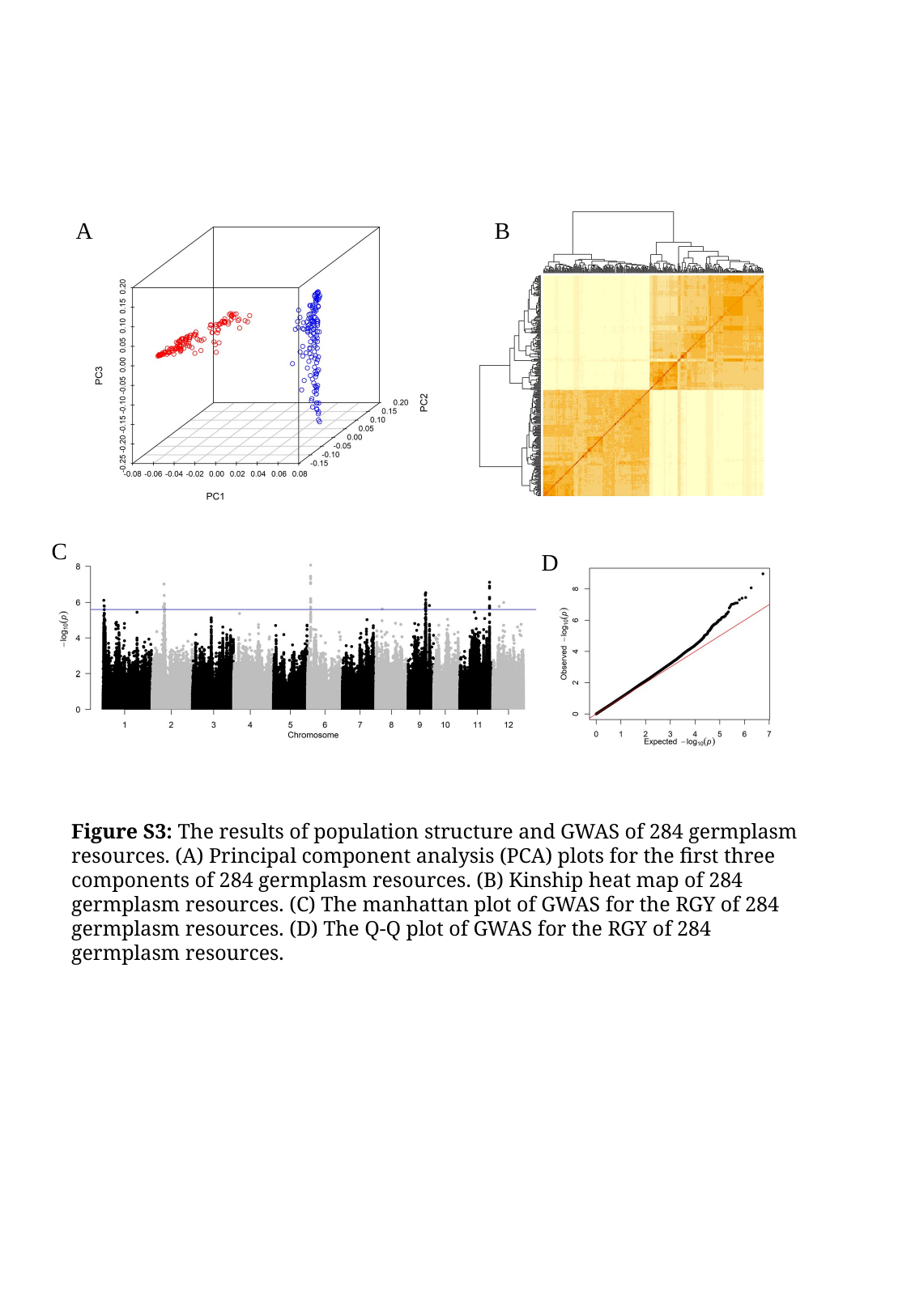

A
B
C
D
Figure S3: The results of population structure and GWAS of 284 germplasm resources. (A) Principal component analysis (PCA) plots for the first three components of 284 germplasm resources. (B) Kinship heat map of 284 germplasm resources. (C) The manhattan plot of GWAS for the RGY of 284 germplasm resources. (D) The Q-Q plot of GWAS for the RGY of 284 germplasm resources.
